# The association between post-traumatic stress-related symptoms, resilience, current stress and past exposure to violence: a cross sectional study of the survival of Quechua women in the aftermath of the Peruvian armed conflict

**DOI:** 10.1186/1752-1505-7-21

**Published:** 2013-10-23

**Authors:** Eliana B Suarez

**Affiliations:** 1 Faculty of Social Work, Wilfrid Laurier University, 120 Duke Street West, Kitchener, Ontario N2H 3W8, Canada

**Keywords:** Resilience, Political violence, Posttraumatic stress, Quechua women, Peru

## Abstract

**Background:**

The long lasting resilience of individuals and communities affected by mass violence has not been given equal prominence as their suffering. This has often led to psychosocial interventions in post-conflict zones being unresponsive to local realities and ill-equipped to foster local strengths. Responding to the renewed interest in resilience in the field of violence and health, this study examines the resilience and post-traumatic responses of Indigenous Quechua women in the aftermath of the political violence in Peru (1980–2000).

**Methods:**

A cross-sectional study examined the relationship between resilience, post-traumatic responses, exposure to violence during the conflict and current life stress on 151 Quechua women participants. Purposive and convenience sampling strategies were used for recruitment in Ayacucho, the area most exposed to violence. The study instruments were translated to Quechua and Spanish and cross-culturally validated. Data was analyzed using hierarchical regression analysis. A locally informed trauma questionnaire of local idioms of distress was also included in the analysis.

**Findings:**

Sixty percent of women (n = 91) were recruited from Ayacucho city and the rest from three rural villages; the mean age was 45 years old. Despite high levels of exposure to violence, only 9.3% of the sample presented a level of symptoms that indicated possible PTSD. Resilience did not contribute to the overall variance of post-traumatic stress related symptoms, which was predicted by past exposure to violence, current life stress, age, and schooling (*R*^*2*^ = .421). Resilience contributed instead to the variance of avoidance symptoms (Stand *β* = −.198, *t* = −2.595, *p* = 0.010) while not for re-experiencing or arousal symptoms.

**Conclusions:**

These findings identified some of the pathways in which resilience and post-traumatic responses interrelate in the aftermath of violence; yet, they also point to the complexity of their relationship, which is not fully explained by linear associations, requiring further examination. Age and gender-sensitive health care is considered critical almost fifteen years after the end of the conflict. The notable resilience of Quechua women—despite exposure to a continuum of violence and social inequalities—also calls for enhanced recognition of women not only as victims of violence but also as complex social actors in the reconstruction of post-conflict societies.

## Background

Many of the victims in contemporary armed conflicts are civilians that often need psychosocial assistance. However, interventions in post-conflict zones frequently are not responsive to local realities, rendering them ill-equipped to foster both local strengths and participation in the social repair process [1,2]. This is due in some part to the fact that the resilience—how individuals survive and thrive despite adversity—of communities and individuals affected by mass violence has not been given equal prominence as their suffering [3]. Resilience has accordingly been called “the next ‘frontier knowledge’ in the field of violence and health” [4].

Conceptualizing resilience in response to mass violence presents several challenges. In those studies that consider resilience a comparative measure of the recovery trajectory of post-traumatic symptomatology, higher resilience is evaluated by observing a lower level or absence of symptoms, in particular, of post-traumatic stress disorder (PTSD) [5]. Other authors measure resilience as an independent construct [6], following perspectives of resilience as a more complex construct than being low in symptoms or symptom-free [7]. This study endorses the view of resilience as a social process resulting from the ability of the individual to survive, and the presence of a social environment that allows for the exercise of this ability [8]. The measurement of resilience represents an additional challenge and several instruments have been developed in response—for example, the Sense of Coherence (SOC) scale [6] and the Connor-Davidson Resilience Scale (CD-RISC) [9]—that have been adapted to multiple contexts, including post-war zones.

The gendered nature of peace has been also less emphasized than the gendered nature of war [10]. The emphasis has been on the marked gender differences in victimization patterns, where women are primarily the victims of sexual and other gender based targeted violence [11] that likely continues well after the conflict has ended [12]. It is less recognized, however, that women also assume important social roles beyond victimhood during war and in the aftermath of conflicts [13]. Women, particularly Indigenous and marginalized women, remain underrepresented in the political governance of post-conflict societies [14]; yet, despite these limitations, women at the margins still exercise agency and contribute to social repair in post-conflict societies [15-17]. The Peruvian armed conflict offers an important example of Indigenous women’s active efforts on survival in the aftermath of war.

### The Peruvian armed conflict and its aftermath

The Peruvian armed conflict lasted from 1980 until 2000, originating in the Andean province of Ayacucho when the rebel group *Sendero Luminoso* [Shining Path] initiated a violent campaign to oust the Peruvian state. The government responded with a fierce counterinsurgency, while also supporting local peasant self-defense units known as *rondas campesinas.* Although some communities defended their perimeters, many were forced to leave their villages and the region. The conflict gradually pervaded other regions, but the highest level of violence occurred in the Andean provinces. *Sendero Luminoso* was shattered in 1992 following the capture of top leaders; however, extreme forms of violence prevailed until 2000. The Peruvian Truth and Reconciliation Commission (*Comisión de la Verdad y Reconciliación,* CVR) was established in 2001. The CVR reported that 69,280 people had disappeared or were killed as a result of the conflict. Approximately 75% of these victims were young and indigenous, mostly of Andean background, who spoke either Quechua or another native language [18], which indicates the disproportionate distribution of death and disappearances by geography, class, and ethnicity [19]. *Sendero Luminoso* was responsible for 54% of the deaths of victims; on the other hand, responsibility for sexual violence was reversed, with *Sendero Luminoso* liable for 11% of reported cases of rape, and the state forces for 85% [18], which makes the prosecution of perpetrators extremely difficult. Indeed, ten years after the final report of the CVR, victims remain disappointed by the ineffectiveness and inaccessibility of the promised reparations.

Estimated prevalence rates of post-trauma symptoms among this population ranged from 12.5% using the ICD classification [20] to 24.7% using the definition of the DSM IV [21]. The extent of sexual violence and other abuses perpetrated toward Quechua women was recognized in the final report of the CVR, as were their struggles to survive while being the sole caretakers of their families and communities. What the CVR’s report fails to recognize is the fact that indigenous Quechua women have been actively involved in the reconstruction process of their communities after the conflict, a failure it shares with the mainstream population. For instance, ethnographic work in post-conflict has highlighted the presence of Quechua women in Ayacucho’s civic society as community organizers, caregivers, and human rights activists [22,23].

### Study conceptual framework and objectives

Galtung’s [24] theory of structural violence explains the role of social arrangements that expose certain individuals or groups to harmful conditions, including extreme health disadvantages. These arrangements are considered structural because they are embedded in the social, political and economic organization of society, and are classified as violent because they cause harm to people [25]. Structural violence theory is particularly relevant to the Peruvian armed conflict, as it reminds us of the importance of studying the legitimization of violence as well as the use of violence—in this case, the historical targeted violence toward the Indigenous population in Peru. An important contribution of the CVR was indeed the identification of high levels of historical discrimination against the Indigenous population as the major underlying cause of the violence [18]. As Laplante and Rivero [26] stated, “before becoming victims of war, they had been victims of historical, social, and economic conditions that deprived them of basic social services such as health.” However, the distress and suffering produced by violence cannot be effectively addressed without further understanding of the simultaneous resilience process [27]. This study expands this understanding by examining the resilience as well as the post-traumatic responses of Quechua women in post-conflict Ayacucho. Furthermore, this study was based on the expressed interest of Quechua women in having their strengths [*fortaleza*] recognized^a^. The objective of this exploratory study was therefore to examine the associations between resilience, past exposure to violence, current life stress, and PTSD-related symptoms among Quechua women in Ayacucho. It was hypothesized that PTSD-related symptoms would be positively associated with current life stress and previous exposure to violence, while inversely associated with resilience.

## Methods

### Participants and procedures

This was a cross-sectional study using structured individual interviews. Face-to-face interviews were conducted throughout June, July and August of 2010, with a sample consisting of 151 Quechua women from the city of Ayacucho and three rural villages: Socos, Maucallaqta, and Ccayarpachi. The study was approved by the University of Toronto Health Sciences Research Ethics Board. Purposive sampling of heterogeneous instances^b^[28] was used as the primary sampling strategy in order to suit the exploratory goals of the study. These purposeful strategies were combined with convenience sampling strategies. The use of these diverse strategies of recruitment precluded the calculation of an exact response rate. The study used flyers and oral presentations at various sites in Ayacucho City to recruit voluntary participants; these sites included food markets, diverse grassroots civic associations such as mother’s clubs, and associations representing the victims and displaced of the conflict. In the three rural villages, the recruitment followed different steps: the researcher first obtained permission from the local authorities, and then posted flyers. The recruitment was largely done, however, by word of mouth. These communities were selected not only due to their geographical diversity, but because of their different experiences during the conflict. For instance, Socos was one of the villages in Ayacucho that promptly developed its own defensive force of “*ronderos*” against the Shining Path. Socos and Maucallaqta are also areas where most residents either never were displaced during the armed conflict, or returned to the same location after the conflict. In contrast, the newly formed ‘*comunidad campesina’* of Ccayarpachi is mainly composed of people displaced from other villages.

Three bilingual (Quechua-Spanish) research assistants and this author conducted the interviews. Once a woman agreed to be interviewed, signed or verbal informed consent—corroborated by the translator/interviewer as witness—was then obtained. Because of the prevailing illiteracy of the study population, the study protocols and survey were administered verbally and constructed in simple terms to facilitate comprehension. Participants were Quechua women aged 25 years or older, who lived in the region of Ayacucho during the conflict—at least part of the time. The age criterion was established to include only women who were at least 10 years old in 1995, the end of the conflict in the area. Table 1 describes the most salient demographic characteristics of the sample.

**Table 1:** Socio-demographics of sample

	***N =*** **151**	**%**	***M (SD)***
**Age**			46.76 (14.5)
**Education**			
No schooling	34	22.5%	
Elementary	65	43.0%	
Secondary	30	19.9%	
Post-secondary	22	14.6%	
**Occupation**			
Survival farming/House	94	62.3%	
Merchant	44	29.1%	
Employee/other	13	8.6%	
**Marital status**			
Single	23	12.4%	
Married/common in law	99	53.5%	
Separated/divorced	5	3.3%	
Widow	24	13.0%	
**Migration status**			
Never moved	69	45.7%	
Returnee	14	9.3%	
Displaced	68	45.0%	
**Residency**			
Urban	91	60.3%	
Rural	60	39.7%	
**Regular participation in civic associations**			
Yes	78	51.7%	
No	73	48.3%	
Income*			
Not enough	54	35.8%	
Enough not sufficient	95	62.9%	
Sufficient	2	1.3%	
**Literacy (Read)**			
Yes	98	64.9%	
No	53	35.1%	
**Living arrangements**			
Living alone	8	5.3%	
Living with others	143	94.7%	

*Enough income covers basic needs such as food and housing, sufficient income covers also primary needs such as health and education. The majority of the sample had no formal job which precluded the calculation of a numerical measure of income.

Measuring instruments were translated, and back translated from English to Spanish and Quechua to assess for semantic and cultural validity. Two of the instruments—Harvard Trauma Questionnaire Part I and Part IV—were already translated and validated within the context of the Quechua-speaking population by Pedersen and colleagues [21]. The other two instruments—the Connor-Davidson Resilience Scale and the Life Stress Questionnaire—were translated and validated following the model of cultural validation proposed by Van Ommeren et al. [29], which triangulates information from translators and key informants with the findings from focus groups with homologous participants. In addition, the survey included the Trauma Questionnaire developed by Pedersen et al [21] based on local idioms of distress (TQ-LID) of the Andean population.

### Study instruments and variables

#### Resilience

The *Connor-Davidson Resilience Scale* (CD-RISC) [9] measured resilience in this study. The CD-RISC defines resilience as “a measure of stress-coping ability that varies with context, age, gender, time, and culture, as well as with different types of adversity.” The CD-RISC consists of 25 items rated on a 5-point range of responses from “not true at all (0)” to “true nearly all the time (4).” Sample items include: “I have at least one close and secure relationship that helps me when I am stressed,” “even when things look hopeless I don’t give up,” “I try to see the humorous side of things when I am faced with problems,” and “I take pride in my achievements.” In this study, the CD-RISC reached adequate internal reliability (Cronbach’s α = .85). The instrument has been translated to several languages; the study used a validated Spanish version of the scale, which was translated to Quechua^c^. A multi-level validation process confirmed the semantic equivalence of the translation. Informants nonetheless observed that some components of resilience within the Andean culture were absent, for instance, cultural identity and reciprocity.

#### General exposure to violence and PTSD-related symptoms

The Harvard Trauma Questionnaire (HTQ) [30] measured general exposure to violence and PTSD-related symptoms in this study. The HTQ is a standardized survey instrument designed to measure exposure to traumatic events and severity of post-trauma symptoms. General exposure to violence (GEV) was measured by Part I of the HTQ. The HTQ-GEV consists of a list of incidents of violence or stressful events that the participant either suffered or witnessed during the years of the conflict. Post-traumatic stress related symptoms (PTSD-R) were measured by Part IV of the HTQ. PTSD-R are defined here as symptoms included in the DSM criteria of PTSD (*n* = 16) that the person reported as “experienced during the last month.” The study used the adapted Spanish/Quechua versions of both the HTQ-GEV and HTQ-PTSD-R, validated for use with the Quechua population by Pedersen et al. [21] [see also 31]. HTQ-GEV scored 1 for events that had been suffered from or witnessed, and 0 for events that had not been suffered from or witnessed. This scoring was applied to all 15 of the items detailing different types of exposure to potentially traumatic events during the conflict, for example, being seriously wounded or nearly dead, tortured, engaged in combat, etc. Tremblay and colleagues [31] reported high internal consistency of the HTQ Part IV of Cronbach’s α = .81 in a sample in the Peruvian highlands. Similarly, the HTQ Part IV exhibited good internal reliability in this study, Cronbach’s α = .87. In addition, the study included a trauma questionnaire based on local idioms of distress (TQ-LID) developed by Pedersen and colleagues [21] based on ethnographic data. Some examples are: “*llaki*” (sorrow) and “*alkansu”* (reached by an evil spirit). The TQ-LID shows high reliability in this study, Cronbach’s α = .87.

#### Current life stress

Current life stress is understood here as recent life events that are perceived by the individual to be stressful. Research in psychosocial aspects of post-traumatic stress indicates that life stressors in the aftermath of trauma may hinder resilience and/or enhance the probability of PTSD-related symptoms [32]; therefore, the inclusion of a measure of current life stress was well justified. *The Life Stress Questionnaire* (LSQ) [adapted from 33] is a list of events that possibly happened in the past year, and their self-assessed stress impact on participants. The original scaling values showed Kendall’s concordance coefficient of *W* = 0.446 (*p* < 0.0005, *N* = 394), meaning that a significant trend of positive agreement in the ranking of stressful events was observed in this sample [33]. Different versions of the LSQ have been widely used in psychosomatic research since the 1960s, for instance, Ahearn and Noble’s [34] study on internally displaced war survivors in Nicaragua. The Spanish version of the scale was translated to Quechua and the new Quechua version was validated by a focus group of homologous participants and feedback from key informants. Some of the 43 events in the LSQ were modified according to relevance to Quechua women. This measure assigns a value of 10–100 for each stressor, rated on the degree to which it has affected the person: greatly, moderately, or slightly. A total score of 300 or above indicates an extremely high level of stress, from 150 to 300, a moderately high level, and under 150 a low stress level.

#### Sociodemographics

Consistent with the purposeful sampling design of the study, a selection of sociodemographic characteristics were incorporated in the questionnaire, such as age, marital status, literacy, education, religion, income, occupation, living in urban or rural areas, living arrangements (alone or with others), migration status, and participation in civic associations. A final open-ended question gave participants the option to further comment on any resilience element that participants would like.

## Results

All data analyses were performed using PASW 18 (Predictive Analysis Software). With the exception of age, a continuous variable, sociodemographic data was classified into categories. The 42-item LSQ mean score was 248.87 (*SD* = 140.30), with a median of 224.00. These scores indicated, on average, a moderately high level of life stress (200–300) for this sample. The global score of the HTQ-PTSD-R was obtained from raw scores following the guidelines of Mollica, McDonald, Masagli and Silove [30], showing a mean of 1.83 (*SD* = 0.45). Using the original cutoff of 2.5, as suggested by Mollica and colleagues [30], only 9.3% (*N* = 14) of the sample show a level of symptoms indicating possible PTSD. The CD-RISC mean score in this study (*M* = 63.99) is lower than the mean score of 80.4 found in the original validation study of the scale in a national community sample in the United States [9], but higher than mean scores found in two clinical samples of PTSD patients: 47.8 and 52.8 [9]. Reliability was assessed with Cronbach’s alpha for all computed variables, but not for HTQ-GEV or LSQ, since it was unsuitable to expect internal consistency within a list of potentially traumatizing or stressful events [35]. Overall, all standardized scales (CD-RISC, HTQ-PTSD-R and TQ-LID) have a *Cronbach’s* α > .80, which indicates adequate reliability to include these scales in the analyses. After assessment of continuous variables for skewness and kurtosis, no data transformations were required.

Participants reported having suffered or witnessed an average of almost 9 violent events during the years of the conflict, for instance, 67% were severely injured or almost killed, 58% were tortured, while the most common event experienced was the violent death of family members and neighbors (83%). Table 2 details the exposure to violence of the study sample.

**Table 2:** **Traumatic events experienced or witnessed as assessed by modified HTQ-GEV (*N*** **= 151)**

***Traumatic event***	***N***	**%**
1. Severely injured or almost killed	101	66.9%
2. Tortured	88	58.3%
3. Combat or armed encounter	81	53.6%
4. Captured or put in prison	82	54.3%
5. Received death threats	94	62.3%
6. Sexually assaulted, battered, abused	47	31.1%
7. Forced to serve in the military/Sendero/rondas*	57	37.7%
8. Forced to kill others	23	15.2%
9. Violent death of family members, relatives	104	68.9%
10. Violent death of friends or neighbours	126	83.4%
11. Any of your relatives/friends disappeared	109	72.2%
12. House/animals/harvest were burnt or stolen	87	57.6%
13. Forced to escape or have passed days starving	108	71.5%
14. Forced to seek refuge in a village nearby	96	63.6%
15. Forced to seek refuge in another city, town	105	69.5%

*Sendero: Rebel group named *Sendero Luminoso* (Shining Path); *Rondas*: Auto defence groups that were formed in some rural communities to fight against *Sendero.*

Pearson correlations (Table 3) show a strong association of CD-RISC with age, HTQ-PTSD and TQ-LID. The last indicates higher resilience for women with lower scores of post-trauma symptoms, as well as for younger women. HTQ-PTSD-R was strongly associated with LSQ, HTQ-GEV, age, and CD-RISC (in this order). This indicates higher levels of PTSD-related symptoms for women with higher levels of current stress, higher exposure to violence during the conflict, older age, and lower levels of resilience. As expected, PTSD-R and TQ-LID were highly correlated *r* = .793 (*p* < 0.001). Considering that both instruments measure post-trauma symptoms, and the recognition of the HTQ as the gold standard measure, only the HTQ was used in the analysis to avoid multi-collinearity.

**Table 3:** Bivariate correlations among variables

***Variables***	***2***	***3***	***4***	***5***	***6***	***M***	***SD***
1.CD-RISC	−.189*	−.123	−.104	−.258*	−.363**	63.99	16.03
2.HTQ-PTSD-R	1	.495**	.498**	.392**	.793**	1.83	0.45
3.HTQ- GEV		1	.523**	.205	.351**	8.68	3.94
4.LSQ			1	.268**	.509**	248.87	140.30
5.AGE				1	.411**	46.76	14.50
6.TQ-LID					1	28.62	7.44

CD-RISC Connor-Davidson Resilience Scale.

HTQ-PTSD-R Harvard Trauma Questionnaire PTSD Related Symptoms.

HTQ-GEV Harvard Trauma Questionnaire General Exposure to Violence.

LSQ Life Stress Questionnaire.

TQ-LID Trauma Questionnaire based on Local Idioms of Distress.

*M* mean; *SD* Standard Deviation.

*p < .05, ** *p* < .01.

Bivariate analyses reveal that overall scores of CD-RISC and PTSD-R were not different for women living in rural or urban areas. Women who participate regularly in civic associations show a higher level of PTSD-related symptoms (*t* = −2.080, *p* < .05), as well as higher resilience (*t* = −3.203, *p* < .001) compared with women who do not. Returnee women also show a trend of simultaneous higher resilience and higher PTSD-R compared to the other two migratory groups.

### Does resilience contribute to PTSD-related symptoms independent of exposure to violence and current life stress?

To test this hypothesis, a multiple linear regression analysis was conducted with the PTSD-R scores as dependent variable and scores of the HTQ-GEV entered in the first block, scores of LSQ in the second block, and the global CD-RISC score entered in the third block to control for HTQ-GEV and LSQ. As predicted, GEV and LSQ show a strong association with the overall scores of PTSD-R and account for 33% of its variance (*R*^*2*^ = .325). In contrast, CD-RISC was not a significant contributor to the variance of PTSD-R (*β* = − 0.117, *t* = −1.718, *p* = 0.088). To further explore co-variants of PTSD-R in this sample, a second regression model was obtained incorporating the socio-demographic variables of level of education, age, occupation, migratory status, marital status, living arrangements, income, urban or rural residency, civic participation, as well as LSQ and HTQ-GEV. Due to the exploratory nature of these associations a stepwise regression model was applied. A backward method was preferred to a forward method to minimize risk of Type II error, due to exclusion of predictors involved in suppressor effects [36]. In the last model (Table 4), LSQ and GEV remained strongly associated with PTSD-R, while age and no schooling were also associated with PTSD-R (*R*^*2*^ *=* .421). The latter indicates that older women and illiterate women who have a higher level of current stress and have experienced higher exposure to general violence during the conflict are likely to experience higher levels of post-traumatic distress.

**Table 4:** Summary of stepwise regression analysis of Post-Traumatic Stress Related-Symptoms (PTSD-R): Total score on degree on exposure to violence (HTQ-GEV), age, current life stress (LSQ) and no-schooling

**Model**		**Stand. *β***	***t *** **value**	***p *** **value**
**Step 17**	Constant		8.9927	.000
*R =* .649	LSQ	.314	2.104	.000
*R*^*2*^ *=* .421	GEV	.251	3.294	.001
Adj*.R*^*2*^ *=* .401	Age	.180	2.410	.017
	No-schooling	.164	2.247	.026

Previous research has indicated that the three clusters of PTSD symptoms, arousal, re-experiencing, and avoidance/numbing symptoms, are either enhanced or hindered differentially by individual and social factors [37,38]. Recent research on First Nations Canadian youth has also examined the differential association of resilience with the different clusters of post-traumatic stress symptoms [39]. The variable HTQ-PTSD-R was therefore transformed into three separate components: arousal, re-experiencing, and avoidance symptoms, following the symptoms classification of the HTQ [30]. The Cronbach alpha coefficients for the new variables were .74, .76 and .73 for arousal, re-experiencing, and avoidance symptoms respectively, achieving enough reliability to enter the analysis. Three separate hierarchical regression analyses were conducted with these new variables as dependent variables, to examine the contribution of CD-RISC, HTQ-GEV, and LSQ on each of the clusters of symptoms. Results indicated that resilience reached statistical significance only as a contributor of the variance of avoidance symptoms, but not for re-experiencing and arousal symptoms. Summarized results of the regression analyses are presented in Table 5.

**Table 5:** Summary of regression analysis for arousal, re-experiencing and avoidance symptoms

	***PTSD-Re-experiencing Symptoms***	***PTSD-Arousal Symptom***	***PTSD- Avoidance Symptoms***
**HTQ-GEV**	Stand.β = .421	Stand.β = .148	Stand.β = .238
	*t =* 5.525	*t* = 1.810	*t* = 2.686
	*p* = .000	*p* = .072	*p* = .000
**LSQ**	Stand.β = .282	Stand.β = .433	Stand.β = .132
	*t* = 3.712	*t* = 5.287	*t* = 1.492
	*p* = .000	*p* = .000	*p* = .138
**CD-RISC**	Stand.β = −.027	Stand.β = −.057	Stand.β = −.198
	*t* = −.405	*t* = −.807	*t* = −2.595
	*p* = .686	*p* = .421	*p* = .010

An additional hierarchical regression was performed to examine the contribution of resilience, general exposure to violence, and life stressors to post-trauma symptoms when these symptoms are measured by local idioms of distress. In this regression analysis, HTQ-PTSD-R was replaced for the TQ-LID as dependent variable. Results show that in this case resilience makes a significant contribution to post-trauma distress, independent of the contributions of life stress and past exposure to violence (Stand *β* = −. 309, *t* = − 4.590, *p* = 0.000). Current life stress was however the major contributor to the variance of TQ-LID (Stand *β* = .418, *t* = 5.362, *p* = 0.000) while GEV (Stand *β* = .089, *t =* 1.134, *p =* 0.259) did not contribute to additional variance. This model accounted for 34% of the variance (*R*^*2*^ = .337) of TQ-LID. However, when demographic variables were entered LSQ remained strongly associated with TQ-LID as well as schooling, living alone, and being married (Table 6), while CD-RISC did not (Stand *β* = −. 131, *t* = − 1.796, *p* = 0.075), indicating the importance of current life stress and social conditions for participants’ post-conflict distress if expressed by local idioms.

**Table 6:** Summary of regression analyses for Local Idioms of Distress (TQ-LID): total score on degree of current life stress (LSQ), age, no-schooling and elementary-school only, married and living alone

**Model**		**Stand. *β***	***t *** **value**	***p *** **value**
**Step 16**	Constant		5.935	.000
*R =* .677	LSQ	.314	7.080	.000
*R*^*2*^ *=* .459	No-schooling	.251	5.329	.001
Adj*.R*^*2*^ *=* .401	Elementary school only	.180	2.708	.008
	Married	−.162	−2.425	.017
	Living alone	.142	2.090	.038

## Discussion

Despite acute exposure to violence during the conflict and higher levels of current life stress, only 9.3% of the sample showed a level of symptoms that indicates potential PTSD. Alongside this, despite cultural limitations in measurement, the average resilience scores of participants were comparable to community samples rather than to samples with a psychiatric diagnosis [9]. The goal of the study was to test theoretical predictions on the long term relationship between resilience, PTSD-related symptoms, exposure to violence and current life stress in the aftermath of conflict. Such a complex relationship, of variables which are themselves evolving and multidimensional, yielded a range of important results that will affect several assumptions within the field. For example, the hypothesized inverse relationship of resilience with overall PTSD-related symptoms was not confirmed; that is, resilience did not attenuate the impact of previous exposure to violence and current life stress on global scores of PTSD related symptoms. The multivariate differences in associations of resilience, previous exposure to violence and current life stress with the three clusters of PTSD symptoms confirmed in this study the multidimensional structure of the construct of PTSD and the limitations of linear associations in explaining those differences. The finding that resilience contributed to the variance of avoidance symptoms, but not to other clusters of PTSD symptoms, may also explain why resilience scores were not associated with overall post-traumatic stress related symptoms and caution about the meaning of these clusters of symptoms in non-western samples.

As listed in the HTQ, some examples of avoidance/numbing symptoms include withdrawing from people, being unable to feel emotions, avoiding activities, having less interest in daily routine, avoiding hurtful thoughts, etc. [30]. Therefore, the social nature of avoidance symptoms inevitably links them with the individuals’ social environment. In addition, past exposure to violence during the conflict (HTQ-GEV)—rather than current stressors (LSQ)—was positively associated with avoidance symptoms, which points to the significance of the “worst event(s)” in this type of post-traumatic responses [40]. Avoidance symptoms are often considered central to the diagnosis of PTSD. Research indicates that avoidance and numbing symptoms are uniquely associated with chronic PTSD [41], with “pervasive disturbance”^d^[42], and with a higher likelihood of reaching the full criteria of PTSD diagnosis [42]. The lower level of avoidance symptoms for resilient women in this study therefore explains how women with overall higher levels of re-experiencing and arousal symptoms appear to be functioning well in several life domains, e.g., active engagement in paid or unpaid work, active participation in civic associations, etc. In other words, they do not seem to be experiencing “persistent disturbance” or impairment. Not surprisingly, women who participate regularly in civic associations show simultaneously a higher level of overall PTSD-related symptoms and higher scores of resilience than women who do not participate. In addition, the fact that only 9.3% of the study participants meet the full criteria of possible PTSD is congruent with a lower occurrence of avoidance symptoms.

It is important to note that the violence experienced by participants could have happened 10–20 years ago and the effect of time may be impacting their responses. In fact, previous studies conducted in 2000 [20] and 2001 [22] in the highlands of Ayacucho reported higher levels of comparable post-traumatic stress symptoms than the current study, which was conducted in 2010, indicating a potential healing effect of passage of time. Of particular relevance to these findings is Davidson and Foa’s [43] suggestion that some traumatic events induce a flood of community support, which may reduce social withdrawal and numbing, which are key features of avoidance symptoms. Research also warns of the delayed development of avoidance symptoms (particularly numbing), which may occur because the community or social response ends or becomes unsupportive [37], or because the person’s own efforts in controlling arousal symptoms fail [44]. In this study, Quechua women experienced some sort of supportive community response via the work of the Peruvian Truth and Reconciliation Commission, NGOs and some government agencies, and most importantly, through their own efforts at creating grassroots organizations of various kinds. While current reparation policies are often considered absent or ineffective [45], and NGOs and government interventions remain without formal evaluation [21], participants in this study appraised their own participation in grassroots organizations as very important for their survival. As participants indicated in the open-ended question of the survey: “*My organization gives me strength*” (G-49), and “*Participating in several organizations and being with my family [help me to survive*]” (C-29). These findings are also congruent with Zraly and Nyirazinyoye’s [17] assertion of the potential therapeutic role of women’s associations in their ethnographic study with women survivors of genocide-rape in Rwanda.

The limitations of the study also caution about the interpretation of the findings. For instance, the use of convenience and purposive sampling strategies means that the sample was not representative of the female populations of Quechua speaking women of the city of Ayacucho and villages of Socos, Maucallaqta and Ccayarpachi, limiting the generalizability of the findings. In addition, the cross sectional design of the study precludes establishing causality between variables. Similar to previous research in the area [21], the study was also unable to include a non-exposed group, due to the extent of the violence within the Quechua population in this region, which precluded the employment of techniques such as propensity scores to control for confounding variables. The small sample size—less than 200—also precluded further analyses such as structural equation modeling, moderator or path analyses [46] which could have been useful to explain reciprocal and/or circular associations between variables. The study did not seek information on traumatic events prior to, or after the armed conflict; therefore, the impact of those events may have confounded the outcomes. In addition, the study is based on self-reported symptoms and not on a clinical diagnosis of PTSD, depression, or other syndromes often presented in comorbidity with PTSD that were not evaluated. Recall of events can be also affected by the distal time when the experiences occurred. Limitations on content validity of the instruments may have also influenced the study findings. Studies in other countries have tended to report lower scores of the CD-RISC than in North America, which indicates cultural differences in the conceptualization of resilience [9]. The validation process of the resilience scale indeed pointed to three local indicators of resilience (cultural identity, capacity to reciprocate, and perception of community support) that were not included in the CD-RISC. Similarly, post-traumatic responses (as defined by PTSD symptoms) do not capture all the expressions of post-trauma distress of the Quechua population, as previously indicated by Pedersen and colleagues [21]. The high correlation of the HTQ-PTSD-R with the TQ-LID indicate the congruency of both measures but their differential associations with other variables pointed to conceptual differences of distress. The study of indigenous resilience using non-indigenous frameworks also suggests cautionary interpretation of the findings [8,39]. The study’s use of face-to-face oral interviews in the participant’s language of preference (Quechua or Spanish) may have minimized the literacy gap of a number of participants and response bias. In fact, participants often commented that they were very interested in the questions related to their resilience, as they had not had other opportunities to reflect on this topic. In this way, the research process may have had an empowering effect for some participants. Despite the limitations, the study is relevant to Quechua women living in post-conflict Ayacucho, therefore contributing to the limited number of studies of women’s resilience in the aftermath of political violence.

The study findings contribute to the existing debate between normalizing and depathologizing symptomatic survivors of wars, or perceiving them as mainly suffering from syndromes of distress, such as PTSD [47,48]. This study substantiates that PTSD-related symptoms *exist* in this sample; however, these symptoms only explain to some extent the distress and the co-existing survival of this otherwise highly functional sample. Additional studies are presenting findings of highly symptomatic civilian victims of war, who are nonetheless displaying functional resilience, for instance in Bosnia [49] and Uganda [50]. The co-existence of suffering and resilience observed in this study is also congruent with the findings of a recent study in Israel, which indicated that at a certain level, cumulative adversity may be simultaneously associated with higher levels of both distress and well-being [51].

The fact that half of the women in this study displayed a high level of social participation while highly symptomatic asserts the protective role of resilience against avoidance symptoms. It also points toward a cautionary interpretation of PTSD symptoms in the context of political violence, particularly when using non-Indigenous views of distress to evaluate Indigenous responses to traumatic events. For instance, in this study, despite being highly correlated, the HTQ, measuring PTSD-related symptoms, and the TQ, a trauma questionnaire based on local idioms of distress, appear to be influenced differently by resilience, exposure to violence, and current life stress, indicating possible etiological differences. Pedersen and colleagues [21] also reported that different predictors influenced both measures, though resilience was not a variable included in their analysis. It is clear that more empirical evidence is necessary to elucidate conclusive findings; in particular, on the differences between these two instruments, and in general, between Indigenous expressions of distress and Western informed measures. Nevertheless, it is undeniable that exposure to long-term mass violence and to enhanced loss of resources is often accompanied by unrelenting distress, as in the case of a number of women in this study, and points to the need for resource-based interventions [5]. In fact, the study shows that it is problematic to talk about “post” traumatic stress, given that stressful experiences of everyday violence (such as poverty, gender-based violence, and discrimination) continue to happen to Quechua women, a situation that appears to enhance the vulnerability to enhanced symptoms of distress for some people, as indicated by the findings of this study. Current life stress and instances of discrimination, such as limited access or no access at all to education, were predictors of enhanced responses of distress irrespective of whether these responses were measured by PTSD related symptoms or local idioms of distress.

The unrelenting exposure to violence affecting Quechua women reflects Galtung’s three concepts of violence: the *events* of direct violence, which trigger the continuous *process* of structural violence up or down, legitimated by permanent *invariant* cultural violence [24]. In this case the armed conflict provided the direct violence towards Indigenous women that perpetuated their experiences of everyday structural violence (poverty, illiteracy, wife abuse), reinforced by the “invariant” and continuous discrimination of Indigenous women in Peru. Indeed, social indicators of structural violence (illiteracy), as well as direct experiences of violence during the conflict and older age were identified as enhancing post-trauma distress in this study. Quechua women are indeed a group persistently targeted by structural violence because of their two social identities—Indigenous and women—which place them at the margins of Peruvian society. For instance, the zones with a majority of indigenous population are also the poorest zones in Peru—with lower levels of income and education than the rest of the country. For instance, the poverty level in Ayacucho (62.6%) is considerably higher than the overall poverty level (34.9%) in the country [52]. In addition, the regional rate of illiteracy in 2009 was 15% (amongst the population 15 years old and above), compared to the national average of 7.6% [52]. Findings of this study, however, indicate that 35.1% (*N* = 53) of the sample was unable to read, and 22.5% (*N* = 34) had never received any formal education, which suggests that disaggregated data placed Indigenous women at greater disadvantage than their male counterparts.

A sequential analysis was precluded by the cross sectional design of the study, but these findings also suggests the presence of delayed onset of PTSD-related symptoms or chronic distress in older participants. Ong and Carter [53] assert that delayed onset can re-occur 10 to 60 years after a war, throughout the ageing process, because of current stressors such as isolation, and new significant losses. Several studies have indeed indicated lifelong mental health consequences of war for women in various post-conflict zones, in particular women who had experienced sexual violence, for instance during WWII [54] or in Bosnia [55]. Attention to delayed responses of distress is therefore crucial, especially in contexts where women experience a continuum of violence from war to peace and where older women are isolated.

Another revealing finding was that the trend of higher resilience of women who had returned to their original communities was accompanied by a higher level of posttraumatic stress related symptoms. Displaced persons received government support to return home after the conflict in Peru [56]. Considering the protective role of resources in the development of resilience [8], it was not surprising that women who had returned to their original locations of residency were identified as displaying higher levels of resilience than women who are displaced or never moved; yet it does not explain the simultaneous higher level of posttraumatic stress related symptoms that contradict previous research findings [57]. Displaced or relocated participants have shown inhibited resilience compared to returnees, which is consistent with findings of the detrimental effects of displacement—for example, Colombia’s forced migrants [58]. The long term impact of forced migration and internal displacement clearly requires further examination.

## Conclusion

The complex and likely reciprocal association between resilience and post-traumatic responses observed in this study contributes to the literature on the long-term consequences of war trauma. The sequelae of suffering in the aftermath of the conflict were undeniable yet resilience was present. Further research would benefit with the use of multiple methodologies and larger samples to deepen the understanding of the role of resilience in post conflict communities. Findings also suggest that screening for risk of delayed onset of symptoms or chronic symptoms will lessen further distress for vulnerable groups of women survivors of war, for example, in this study, older Quechua women. Mental health services should be sensitive to social inequalities, in particular gender disparities, and bring local resources into play for healing and recovery in order to be effective. As Nguyen and colleagues stated “the search for psychological well-being and justice are not mutually exclusive” [59]. The manifest limitations on the content validity of instruments in this study indicate the need for inclusion of local knowledge and practices in the study of conflict and health. The study findings emphasize the simultaneous presence of resilience and post-traumatic responses, the targeted occurrence of traumatic events, and the importance of local contexts in explanatory frameworks of war trauma. The noteworthy association of civic participation and activism with the resilience of Quechua women—despite exposure to a continuum of violence and social inequalities—also calls for enhanced recognition of women in the aftermath of conflicts, looking beyond their experiences of victimhood to acknowledge their multiple roles as key contributors to the social reconstruction process in post conflict. Moreover, considering that 25 to 50% of post-conflict countries experience renewed conflict [60], and so far local civil societies have had the most decisive role against this reoccurrence [61], it is essential to understand better how individuals and communities survive and protect themselves in the aftermath of mass violence.

## Endnotes

^a^Personal communications from informal meetings realized in preliminary field trip to Ayacucho in June 2009 with Quechua women members of ANFASEP.

^b^Purposive sampling involves “defining the characteristics of the persons, settings, treatments, or outcomes to which you want to generalize” [28]. It can be of typical or heterogeneous instances—depending on if you want to define a “typical” person/outcome, or want a sample, which is “heterogeneous,” and not a typical instance at all.

^c^Further questions about the CD-RISC and its availability should be directed to the authors of the scale at http://jonathan.davidson@duke.edu.

^d^In contrast to symptom profiles of “no disturbance” and/or “intermediate disturbance,” “pervasive disturbance” was defined as experiencing a high number of PTSD symptoms (< 12), with a large proportion of numbing symptoms that interfere with the individual’s life or activities for more than 60 months [42].

## Abbreviations

CVR: *Comisión de la Verdad y Reconciliación* (Peruvian Truth and Reconciliation Commission); DSM IV: Diagnostic and statistical manual of mental disorders - IV Edition; ICD: International classification of disorders; PTSD: Post-traumatic stress disorder.

## Competing interests

The author declares that there are no competing interests.
